# *MYCT1-TV*, A Novel *MYCT1* Transcript, Is Regulated by c-Myc and May Participate in Laryngeal Carcinogenesis

**DOI:** 10.1371/journal.pone.0025648

**Published:** 2011-10-05

**Authors:** Shuang Fu, Yan Guo, Hong Chen, Zhen-Ming Xu, Guang-Bin Qiu, Ming Zhong, Kai-Lai Sun, Wei-Neng Fu

**Affiliations:** 1 Department of Medical Genetics, China Medical University, Shenyang, People's Republic of China; 2 Department of Central Laboratory, School of Stomatology, China Medical University, Shenyang, People's Republic of China; 3 Department of Otolaryngology, The 463 Hospital of PLA, Shenyang, People's Republic of China; 4 Department of Clinical Laboratory, No. 202 Hospital of PLA, Shenyang, People's Republic of China; University of Saarland Medical School, Germany

## Abstract

**Background:**

*MYCT1*, a putative target of c-Myc, is a novel candidate tumor suppressor gene cloned from laryngeal squamous cell carcinoma (LSCC). Its transcriptional regulation and biological effects on LSCC have not been clarified.

**Methodology/Principal Findings:**

Using RACE assay, we cloned a 1106 bp transcript named Myc target 1 transcript variant 1 (*MYCT1-TV*) and confirmed its transcriptional start site was located at 140 bp upstream of the ATG start codon of *MYCT1-TV*. Luciferase, electrophoretic mobility shift and chromatin immunoprecipitation assays confirmed c-Myc could regulate the promoter activity of *MYCT1-TV* by specifically binding to the E-box elements within −886 to −655 bp region. These results were further verified by site-directed mutagenesis and RNA interference (RNAi) assays. *MYCT1-TV* and *MYCT1* expressed lower in LSCC than those in paired adjacent normal laryngeal tissues, and overexpression of *MYCT1-TV* and *MYCT1* could inhibit cell proliferation and invasion and promote apoptosis in LSCC cells.

**Conclusions/Significance:**

Our data indicate that *MYCT1-TV*, a novel *MYCT1* transcript, is regulated by c-Myc and down-regulation of *MYCT1-TV/MYCT1* could contribute to LSCC development and function.

## Introduction

Laryngeal squamous cell carcinoma (LSCC) is the second main upper respiratory tract tumor behind lung cancer in incidence and mortality rates [1], and represents the vast majority (approximately 96%) of laryngeal malignancies [2]. The morbidity of LSCC shows an increasing trend in China, especially in the northeast part. The etiology of LSCC is considered to be multifactorial. The main predisposing factors are tobacco and alcohol abuse [3]. Despite many advances achieved in the diagnosis and treatment of the disease, its overall survival rate has remained unchanged (at approximately 35–70%) over the past several decades [1]. Therefore, it is necessary to develop new diagnostic and therapeutic targets for LSCC.

Although much work is presently focused on the research about the relationship between LSCC and oncogenes, such as *BCL2* [4], *c-Myc* [4], [5] and *EGFR* [6], or tumor suppressor genes (TSGs), such as *P53* [7], *Rb* [8], *P16* [8] and *P21* [8], no information about gene cloning related to laryngeal carcinoma is reported except human *Myc target 1* (*MYCT1*) [9].

*MYCT1* (Gene ID: 80177) is a novel candidate TSG cloned using in silicon hybridization and molecular methods from LSCC by our team, which was previously named *MTLC* (c-Myc target from laryngeal cancer cells, GenBank accession No. AF_527367). The full length of this gene is about 21 kb. It contains two exons and produces a 1006 bp transcript coding a protein with 235 amino acid residues. Our previously study demonstrated that *MYCT1* existed in various human tissues and was down-regulated in gastric carcinoma tissues. The 5′ flanking sequence of *MYCT1* contains two binding sites of oncoprotein *c-Myc* [9], [10]. But whether it is also down-regulated in LSCC or is really regulated by *c-Myc* and its biological effects on LSCC have not been systematically analyzed. In this present work, we have studied this candidate *c-Myc* target gene in greater detail. We cloned a new *MYCT1* transcript variant (*MYCT1-TV*, GenBank accession No. GU997693), confirmed its transcriptional start site (TSS) for the first time, identified a 230 bp region that mediated basal transcription by two E-box elements and explored the role of *MYCT1* and its variant in the development and aggression of LSCC by comparing the expression or biological characteristics between *MYCT1* and its isoform in LSCC tissues or cells, which will lay a foundation for the further exploration on understanding the function of *MYCT1* in c-Myc regulatory subnetwork and provide us a novel LSCC diagnostic and therapeutic target for the future study.

## Results

### Cloning and characterization of *MYCT1-TV*

**Figure 1:**
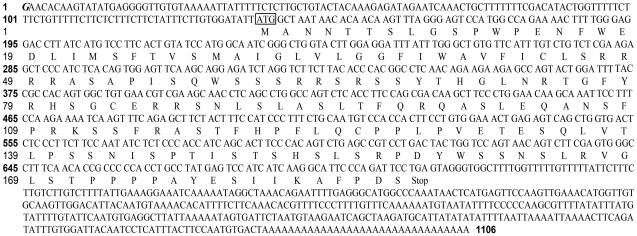
cDNA characteristics of *MYCT1-TV*. cDNA and putative amino acid sequences for *MYCT1-TV*. The 1106 bp cDNA, obtained by 5′ and 3′ RACE, contains a putative open reading frame that encodes a polypeptide of 187 amino acid residues. Transcriptional start site is marked in bold and italic. Initiator codon ATG is boxed.

**Figure 2:**
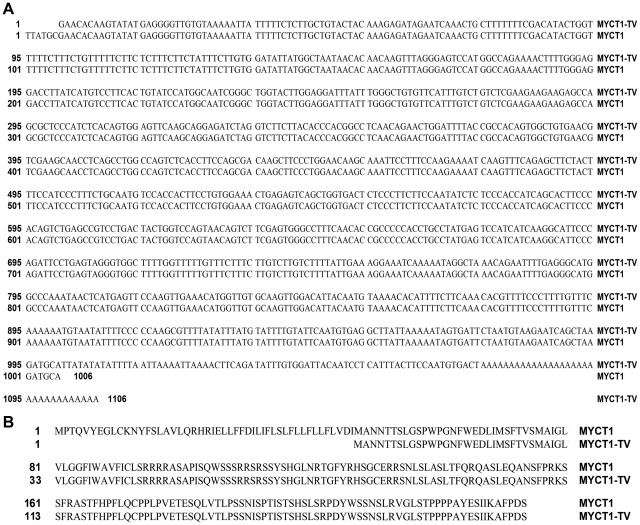
Comparison of MYCT1-TV and MYCT1. **A.** The full-length cDNA clones of *MYCT1-TV* and *MYCT1* are 1106 bp and 1006 bp, respectively. *MYCT1-TV* has a shorter 5′ flanking sequence and a longer 3′ flanking sequence compared to *MYCT1*. **B.** The amino acids sequence comparison of MYCT1-TV and MYCT1.

we obtained a novel 1106 bp transcript variant of *MYCT1* named myc target 1 transcript variant 1 (*MYCT1-TV*, GenBank accession No. GU997693) using RACE and cDNA cloning methods. Our sequencing results indicated a potential *MYCT1-TV* TSS was located at 140 bp upstream of the ATG start codon of *MYCT1-TV* (Fig. 1). Meanwhile, the only TSS we confirmed was also located at 12 bp downstream of the start nucleotide of the published *MYCT1* mRNA sequence (GenBank accession No. AF_527367, Fig. 1). *MYCT1-TV* has a 140 bp 5′ untranslated leader and a 370 bp 3′ untranslated region with a 32 bp poly (A) tail. As predicted using ExPASy proteomics server, the 564 bp open reading frame codes for a putative protein of 187 amino acid residues with a predicted molecular mass of 20835Da and pI 10.26 (Fig. 1). Using NCBI blast, we compared the protein and nucleotide sequences of MYCT1-TV with those of MYCT1 (GenBank accession No. AF_527367). The analysis results revealed that *MYCT1-TV* cDNA has a shorter 5′ flanking sequence and a longer 3′ flanking sequence than *MYCT1* (Fig. 2A). MYCT1-TV protein has a shorter 48 amino acid N-terminus than MYCT1 protein and the other 187 amino acid protein of them are much the same (Fig. 2B). ExPASy proteomics server analysis demonstrated the shorter 48 amino acid protein contains no obviously structural or functional motif.

### Identification and analysis of *MYCT1-TV* promoter region

**Figure 3:**
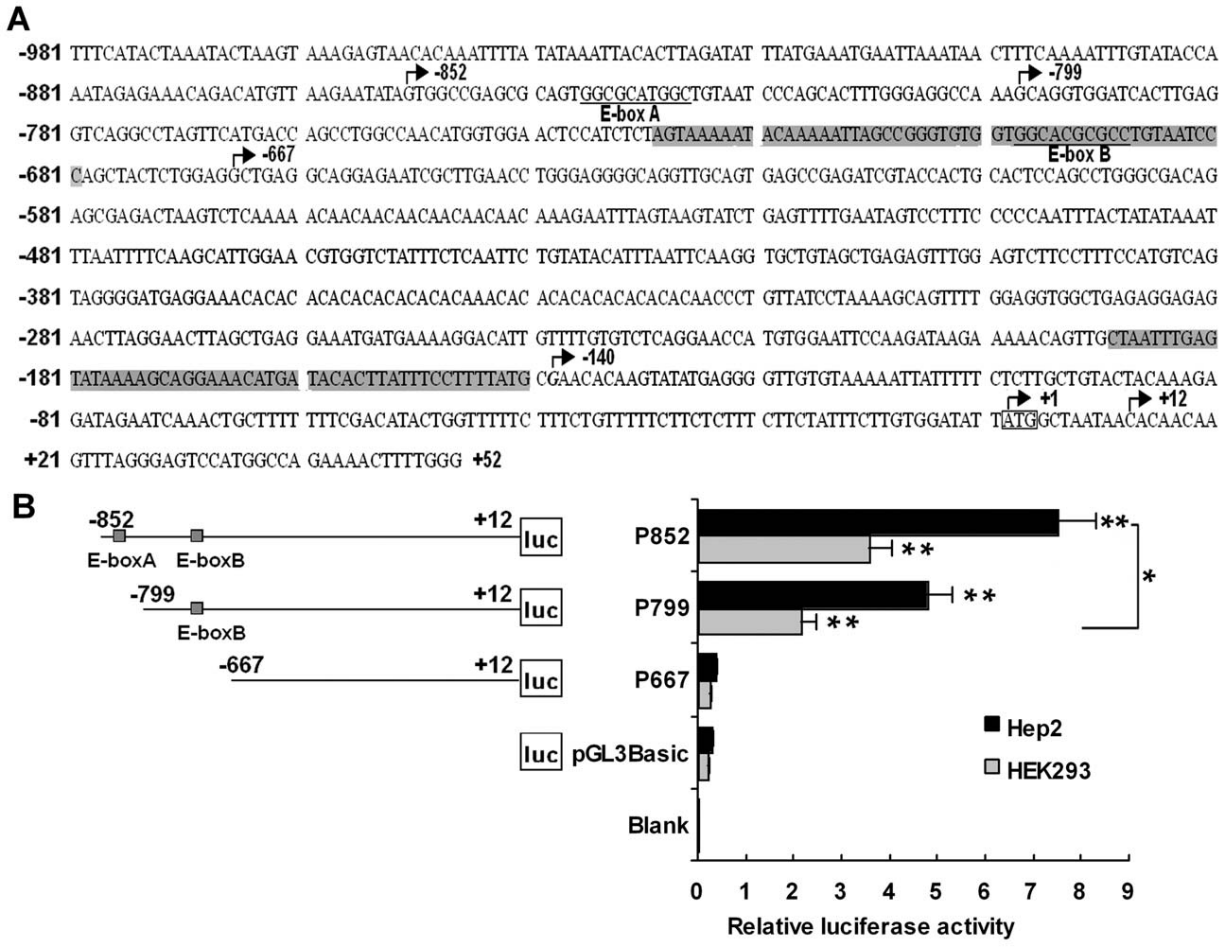
Identification and functional characterization of human *MYCT1-TV* promoter. **A.** Nucleotide sequence of the promoter region (from −981 to +52) of human *MYCT1-TV*. The putative binding sites for transcriptional factors are underlined and markded as E-box A and B. The transcriptional start site is marked in bold and italic at −140 bp. The start nucleotide of the published *MYCT1* mRNA sequence is marked in bold at −152 bp. The positions of putative core promoter sequences are marked with gray color. The numbering of the nucleotides starts at initiator codon ATG (+1) which are boxed. **B.** Analysis of human *MYCT1-TV* promoter activities detected by luciferase assay. Hep2 (black bars) or HEK293 (gray bars) cells are transiently transfected with 0.8 µg of the deletion constructs together with 16 ng pRL-TK. The relative activities of a series of deletion constructs are determined by luciferase assay (**, p<0.01; *, p<0.05).

Using web software BDGP, Promoter 2.0 and Promoter Scan, we analyzed the proximal 1033 bp (−981/+52) promoter sequence of *MYCT1-TV* and found two core promoter sequences in this region (Fig. 3A). In order to determine which region play an important role in promoter activity, we generated a number of consecutive 5′ deletions of *MYCT1-TV* promoter (Fig. 3B). The generated fragments were cloned into pGL3-Basic and transiently co-transfected into Hep2 and HEK293 cells along with pRL-TK. Luciferase results revealed a 7.26-fold increased transcriptional activity of P852 as compared to the empty vector in Hep2 cells, indicating that we obtained an active *MYCT1-TV* promoter (p<0.01, Fig. 3B). Removal of 53 bp at 5′ end from P852 caused a 2.76-fold decrease in luciferase activity (p<0.05, Fig. 3B). However, a further deletion of 132 bp at 5′ end from P799 caused a drastical drop about 7.16-fold of *MYCT1-TV* promoter activity compared to P799 (p<0.01, Fig. 3B). The similar results were also obtained in HEK293 cells (Fig. 3B). These results suggested that the basal regulatory elements important for maximal promoter activity are present between −852 bp and −667 bp region, and P852 (−852/+12) acts as the proximal promoter.

### Importance of two E-boxes in *MYCT1-TV* promoter activity

**Figure 4:**
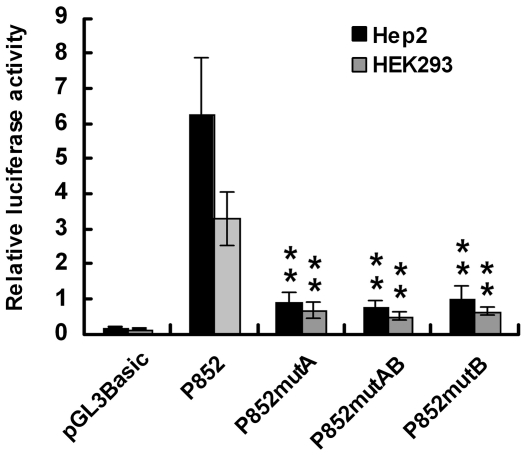
The transcriptional activity of the mutant *MYCT1-TV* core promoter. Promoter constructs pGL3-Basic, P852-mutA, P852-mutB and P852-mutAB were cotransfected with pRL-TK into Hep2 (black bars) or HEK293 (gray bars) cells, respectively by Lipofectamine 2000 Reagent. Luciferase activity was measured at 48 h after transfection (**, p<0.01).

C-Myc is known as a transcription factor through forming heterodimers with its binding partner Max specifically binding to a canonical consensus DNA sequence CACGTG, termed the E-box [11]. And it has also been reported to bind to several other noncanonical DNA motifs, such as CATGTG, CATGCG, CACGCG, CACGAG, and CAACGTG [12]–[14]. In luciferase assay, we confirmed the promoter region between −852 bp and −667 bp was important for basal regulatory elements binding. And two noncanonical E-box elements, CGCATG (the reverse complement of CATGCG) and CACGCG, as we reported before [9], were just present in this region (Fig. 3A). To analyze whether these putative c-Myc/Max-binding sites mediate transcriptional activity of *MYCT1-TV* core promoter, we introduced three point mutations, P852-mutA, P852-mutB and P852-mutAB. Luciferase assay showed that compared to the wide-type promoter P852 in Hep2 cells, mutated only E-box A (P852-mutA) or B (P852-mutB) or mutated both E-box A and B (P852-mutAB) caused a 5.40-fold, 5.28-fold or 5.52-fold reduction of transcriptional activity (p<0.01, Fig. 4). And there were no significant difference between these mutants (p>0.05, Fig. 4). Similar results were also obtained in HEK293 cells (Fig. 4). These results indicated that both of the identified motifs are essential for the basal activity of *MYCT1-TV* core promoter.

### Regulation of *MYCT1-TV* promoter by c-Myc

**Figure 5:**
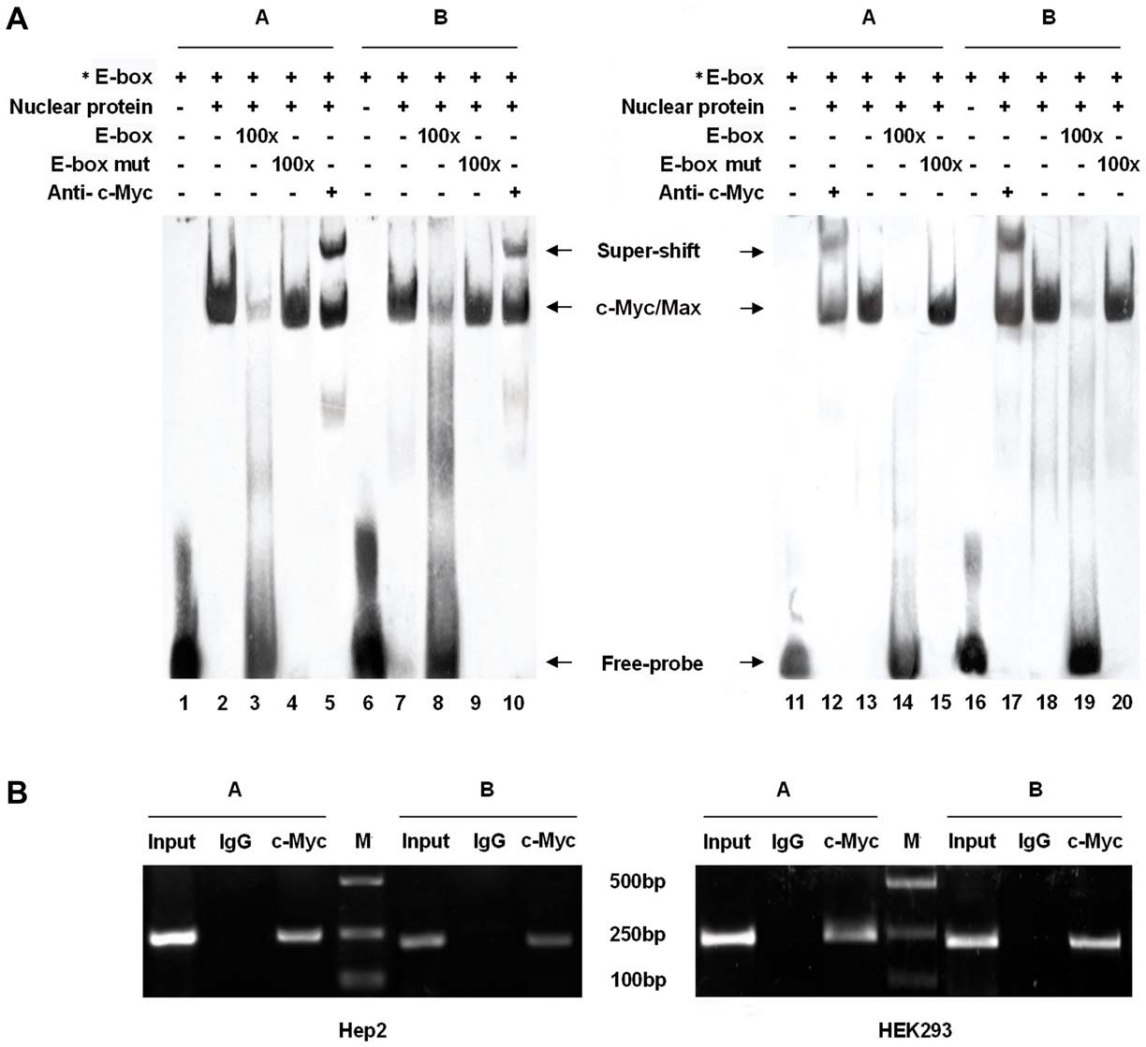
A role for E-box sites in basal human *MTCT1-TV* promoter activity. **A.** Binding of *MYCT1-TV* E-box sites to c-Myc in vitro detected by EMSAs. The symbol “ * ” means the oligonucleotides labled by biotin. Lanes 1 to 10 stand for the results from Hep2 cells and Lanes 11 to 20 the results from HEK293 cells. Lanes 1, 6, 11 and 16 represent biotin-labled oligonucleotides. Lanes 2, 7, 13 and 18 represent each probe incubated with nuclear extracts. Lanes 3, 8, 14 and 19 represent each probe incubated with a 100-fold excess of the unlabeled competitor oligonucleotides. Lanes 4, 9, 15 and 20 represent each probe incubated with a 100-fold excess of the unlabeled mutant competitor oligonucleotides. Lanes 5, 10, 12 and 17 represent the EMSA results in the presence of anti-c-Myc antibody. The experiments were repeated three times. **B.** Binding of E-box sites to c-Myc in vivo detected by ChIP. The Input lanes correspond to PCR products derived from chromatin prior to immunoprecipitation. The IgG lanes correspond to PCR products containing chromatin immunoprecipitated with antibodies against control IgG. The c-Myc lanes correspond to PCR products containing chromatin immunoprecipitated with antibodies against c-Myc. M indicates DNA 2000 marker. The 242 bp PCR product of c-Myc A or the 215 bp PCR product of c-Myc B is obtained corresponding to the sequence either E-box A or B binding site of the *MYCT1-TV* promoter. Results of Hep2 cells shown in the left figure are in line with those of HEK293 cells in the right one.

To assess the importance of E-box elements for c-Myc/Max binding in vitro, we performed EMSA using biotin-labeled, synthetic double-stranded oligonucleotides corresponding to the E-box A and B. These probes were incubated with the nuclear extracts isolated from Hep2 or HEK293 cells. As shown in Fig. 5A (lanes 2, 7, 13 and 18), both oligonucleotides specific to E-box A and B were retarded significantly by cell nuclear proteins. Quantitative competitions for bindings were found at the presence of a nearly 100-fold excess of unlabeled oligonucleotides (lanes 3, 8, 14 and 19), whereas no competitions were observed with a nearly 100-fold excess of unlabeled mutant oligonucleotides (lanes 4, 9, 15 and 20). Furthermore, the bands were supershifted by specific c-Myc antibodies (lanes 5, 10, 12 and 17). The interaction between c-Myc/Max and *MYCT1-TV* promoter was further confirmed by ChIP assay. Using primers to amplify both c-Myc/Max binding regions of *MYCT1-TV* promoter, we observed strong PCR products based on the precipitate by c-Myc antibody from Hep2 and HEK293 cells, but not from negative control immunoprecipitations using anti-rabbit IgG antibody (Fig. 5B).

### Reduced *MYCT1-TV* promoter activity with silencing of c-Myc

**Figure 6:**
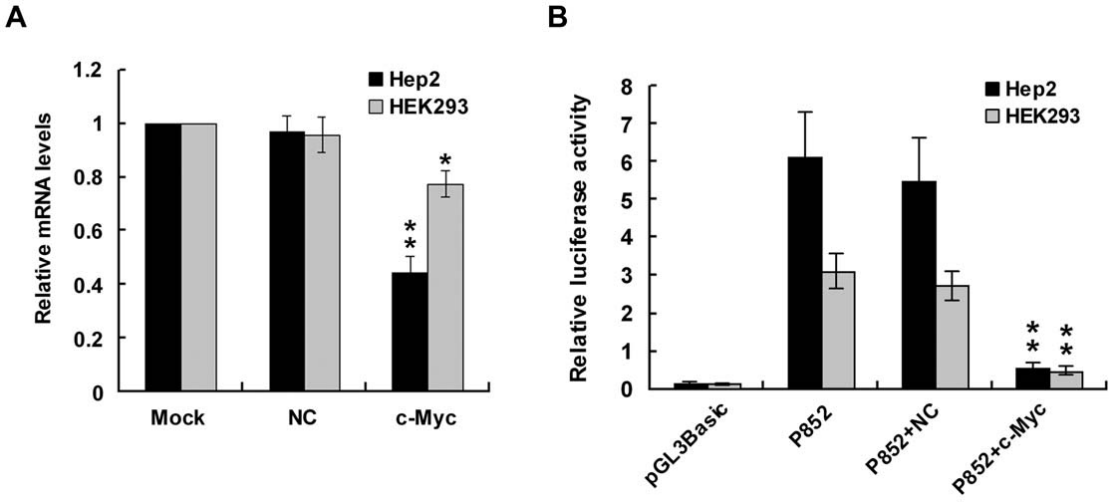
Silencing of endogenous c-Myc reduces *MYCT1-TV* promoter activity. **A.** Detecting of c-Myc expression level in cells by qRT-PCR. The expression of GAPDH was used as an internal control. Hundred nanogram of no siRNA (Mock), nonspecific control siRNA (NC) or c-Myc specific siRNA (c-Myc) were transfected into Hep2 (black bars) or HEK293 (gray bars) cells. **B.** Luciferase activity of *MYCT1-TV* promoter after transfected with c-Myc siRNA. Luciferase activity was measured in Hep2 (black bars) or HEK293 (gray bars) extracts 48 h after transfection. pGL3-Basic, cells cotransfected with pGL3-Basic and pRL-TK; P852, cells cotransfected with P852 and pRL-TK; P852+NC, cells cotransfected with P852, NC siRNA and pRL-TK; P852+c-Myc, cells cotransfected with P852, c-Myc siRNA and pRL-TK. Data are indicated as the means ± SEM of three independent experiments.

To further confirm the role of c-Myc/Max in regulation of *MYCT1-TV* promoter activity, we silenced c-Myc translation via RNA interference. The efficieny of the siRNAs directed against c-Myc was confirmed by qRT-PCR. As compared to the Mock control (no siRNA) in Hep2 and HEK293 cells, transfection of c-Myc specific siRNA effectively knocked down c-Myc mRNA levels (p<0.05, Fig. 6A) whereas transfection of nonspecific control siRNA (NC siRNA) showed no evidence of silencing (p>0.05, Fig. 6A). We then cotransfected the luciferase reporter plasmid P852 containing *MYCT1-TV* promoter together with siRNA. Compared with NC siRNA group, luciferase activity of P852 displayed significant reduction after transfected with c-Myc siRNA (p<0.01, Fig. 6B). Thus, c-Myc can strongly enhance transcriptional activity of *MYCT1-TV* promoter.

### Extensive expression of *MYCT1-TV* in human cells

**Figure 7:**
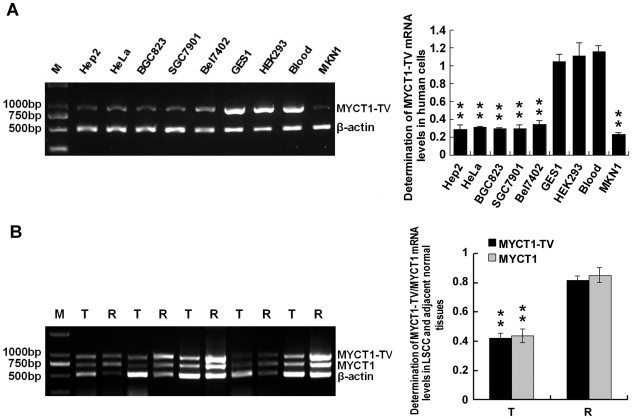
Expression of *MYCT1-TV* and *MYCT1* in human cells and tissues. **A.** *MYCT1-TV* mRNA levels in human cells. The gray-scale ratios of *MYCT1-TV* to β-actin mRNA levels in Hep2, HeLa, BGC823, SGC7901, Bel7402, GES1, HEK293, human blood and MKN1 cells are 0.2890±0.0521, 0.3113±0.0138, 0.2985±0.0130, 0.2964±0.0427, 0.3512±0.0407, 1.0522±0.0808, 1.1159±0.1467, 1.1641±0.0665 and 0.2348±0.0147, respectively. **B.** *MYCT1-TV* and *MYCT1* mRNA levels in LSCC and paired adjacent normal laryngeal tissues. PCR produce a 929 bp DNA fragment for *MYCT1-TV*, a 726 bp DNA fragment for *MYCT1* and a 511 bp DNA fragment for β-actin. The gray-scale ratios of *MYCT1-TV* to β-actin mRNA levels (black bars) in LSCC and paired adjacent normal laryngeal tissues are 0.4172±0.0324 and 0.8073±0.0478, and the counterpart gray-scale ratios of *MYCT1* to β-actin mRNA levels (gray bars) are 0.4304±0.0304 and 0.8416±0.0499. M, T and R indicate DNA marker, LSCC tumor tissue and paired adjacent normal tissue, respectively.

To explore whether *MYCT1-TV* is widely expression or specific expression in human cells, we performed RT-PCR in Hep2, HeLa, BGC823, SGC7901, MKN1, Bel7402, GES1 and HEK293 cells compared with normal human blood (positive control). The result showed that the *MYCT1-TV* extensively expressed in all cells examined, but *MYCT1-TV* mRNA levels in normal cells of GES1 and HEK293, which shared no difference with normal human blood (p>0.05, Fig. 7A), were significantly higher than those in tumour cells of Hep2, HeLa, BGC823, SGC7901, Bel7402 and MKN1 (p<0.01, Fig. 7A). There was also no significant difference in *MYCT1-TV* mRNA levels among the tumour cells above (p>0.05, Fig. 7A).

### Reduced expression of *MYCT1-TV* or *MYCT1* in LSCC tissues

Since *MYCT1* was cloned from LSCC and down-regulated in gastric carcinoma tissues [9], [10], we are interested in exploring whether it is also down-regulated in LSCC and existed the expression differences between *MYCT1-TV* and *MYCT1* in LSCC. RT-PCR results showed that both *MYCT1-TV* and *MYCT1* mRNA levels in LSCC tissues were significantly lower than that in paired adjacent normal laryngeal tissues (p<0.01, Fig. 7B). Moreover the mRNA expression levels of these two transcripts showed no significant difference in LSCC or paired adjacent normal laryngeal tissues (p>0.05, Fig. 7B).

We assessed the expression levels of *MYCT1-TV*/*MYCT1* with respect to clinical characteristics (age, sex and cancer stage). No differences were identified in mRNA levels of *MYCT1-TV*/*MYCT1* with respect to patient age, sex, or patient clinical stage (p>0.05, Table 1).

**Table 1:** Analysis of the relationship between *MYCT1-TV*/*MYCT1* and clinical characteristics (Gray-scale ratio, mean ± SEM).

		mRNA levels	
Characteristic	Response	*MYCT1-TV*	*MYCT1*
Age	<62 y	0.4367±0.0562	0.4480±0.0524
	≥62 y	0.3995±0.0361	0.4144±0.0143
		P = 0.936	P = 0.789
Sex	Male	0.4438±0.0425	0.4433±0.0372
	Female	0.6695±0.1051	0.3940±0.0558
		P = 0.069	P = 0.405
Cancer stage	I	0.5795±0.0954	0.4755±0.0730
	II	0.3889±0.0518	0.4872±0.0843
	III	0.3977±0.0776	0.4175±0.0597
	IV	0.3695±0.0400	0.3875±0.0453
		P = 0.123	P = 0.695

### Function of *MYCT1-TV* and *MYCT1* in Hep2 and HEK293 cells

**Figure 8:**
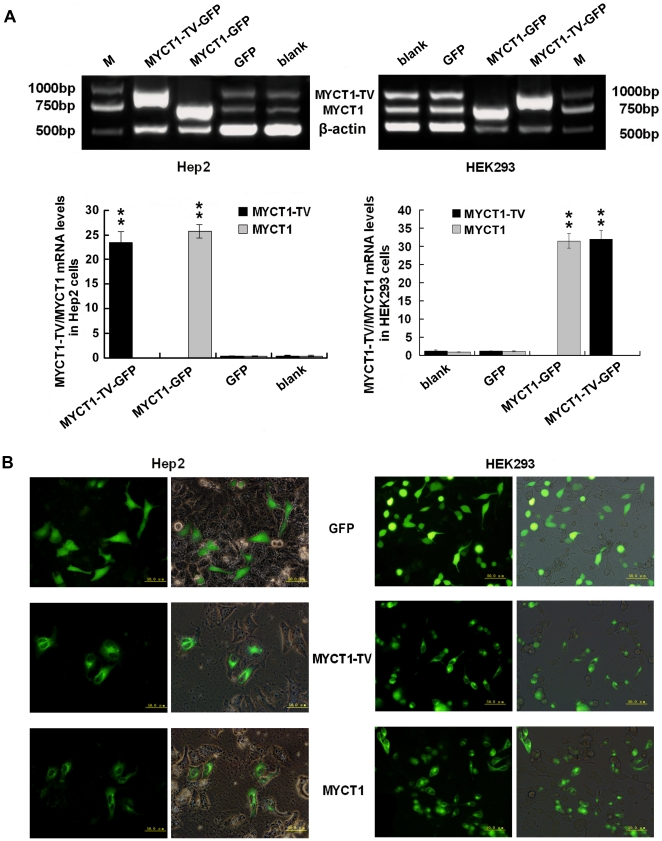
Transfection of *MYCT1-TV* and *MYCT1* in Hep2 and HEK293 cells. **A.** mRNA levels of *MYCT1-TV/MYCT1* in Hep2 and HEK293 cells transfected with MYCT1-TV-GFP/MYCT1-GFP. PCR produce a 929 bp DNA fragment for *MYCT1-TV*, a 726 bp DNA fragment for *MYCT1* and a 511 bp DNA fragment for β-actin. In Hep2 cells, the gray-scale ratios of *MYCT1-TV* to β-actin mRNA levels (black bars) in blank, GFP and MYCT1-TV-GFP groups are 0.4735±0.0335, 0.4315±0.0303 and 23.5188±2.0896, and the gray-scale ratios of *MYCT1* to β-actin mRNA levels (gray bars) in blank, GFP and MYCT1-GFP groups are 0.4157±0.1080, 0.4242±0.0658 and 25.7520±1.3244, respectively. In HEK293 cells, the counterpart gray-scale ratios of *MYCT1-TV* to β-actin mRNA levels are 1.3071±0.2223, 1.2523±0.1002 and 32.0339±2.2903, and the counterpart gray-scale ratios of *MYCT1* to β-actin mRNA levels are 0.9950±0.0725, 1.1448±0.1346 and 31.5161±1.9808, respectively. **B.** Transfection efficiency and expression of MYCT1-TV/MYCT1 protein in Hep2 and HEK293 cells. Transfection efficiency and expression of MYCT1-TV/MYCT1 protein in GFP, MYCT1-TV-GFP and MYCT1-GFP groups are revealed by contrast and fluorescence microscopy under the same phase. M, DNA marker; blank, control cells before transfection; GFP, control cells after transfection with GFP only; MYCT1-TV-GFP, cells transfected with MYCT1-TV-GFP; MYCT1-GFP, cells transfected with MYCT1-GFP (**, p<0.01).

To elucidate the function of *MYCT1-TV* and *MYCT1* in human cells, MYCT1-TV-GFP and MYCT1-GFP expression vectors were generated and transiently transfected into Hep2 and HEK293 cells. Forty-eight hours after transfection, Hep2 cells in MYCT1-TV-GFP and MYCT1-GFP groups showed a significant increase at mRNA levels compared to those in blank and GFP groups (p<0.01, Fig. 8A). MYCT1-TV and MYCT1 protein expression were detected by confocal immunofluorescence microscopy (Fig. 8B). These results revealed that the transfection is successful. Similar results were also obtained in HEK293 cells (Fig. 8A and B).

**Figure 9:**
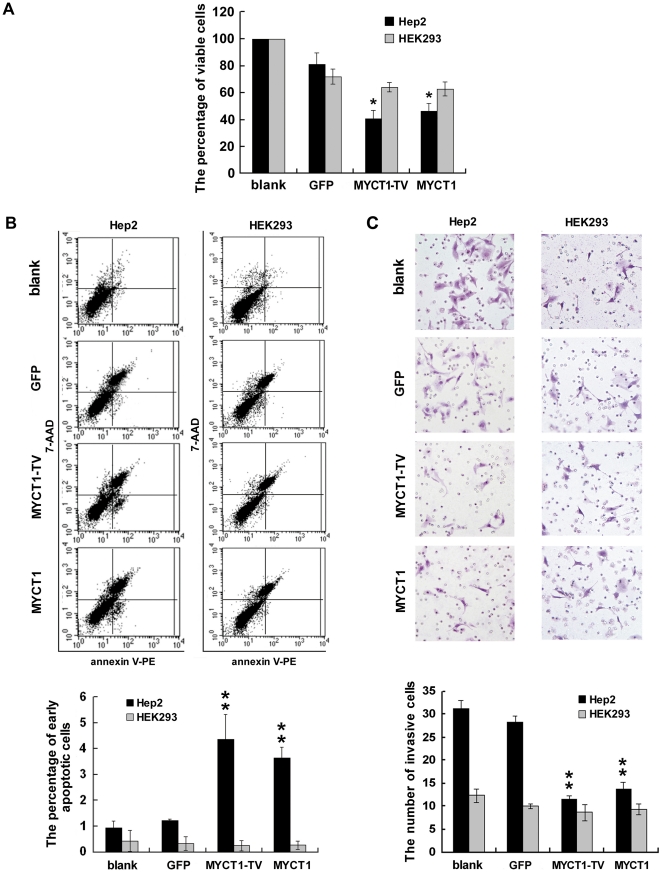
Function of *MYCT1-TV* and *MYCT1* in Hep2 and HEK293 cells. The proliferation of Hep2 and HEK293 cells. **B.** The apoptosis of Hep2 and HEK293 cells. **C.** The invasive ability of Hep2 and HEK293 cells. Blank, control cells before transfection; GFP, control cells after transfection with GFP only; MYCT1-TV, cells transfected with MYCT1-TV-GFP; MYCT1, cells transfected with MYCT1-GFP.

Cell viability assay showed that the proportion of viable cells in MYCT1-TV-GFP or MYCT1-GFP group was significantly fewer than that in GFP group (p<0.05, Fig. 9A). The percentages of viable cells in MYCT1-TV-GFP, MYCT1-GFP and GFP groups relative to that in blank group were 41.46±6.00%, 46.91±5.56% and 81.27±8.61%, respectively. In addition, there was no significant difference between MYCT1-TV-GFP and MYCT1-GFP groups (p>0.05, Fig. 9A). However, similar results were not obtained in HEK293 cells, and the counterparts were 64.47±3.40%, 63.14±5.08% and 72.29±5.62% (p>0.05, Fig. 9A).

Cell apoptosis assay indicated that the proportion of early apoptotic Hep2 cells was significantly higher in MYCT1-TV-GFP or MYCT1-GFP group than those in blank and GFP groups (p<0.01, Fig. 9B). The percentages of early apoptotic Hep2 cells in blank, GFP, MYCT1-TV-GFP and MYCT1-GFP groups were 0.92±0.25%, 1.19±0.08%, 4.37±0.96% and 3.63±0.43%, respectively. In addition, there was no obvious difference between MYCT1-GFP-TV and MYCT1-GFP groups (p>0.05, Fig. 9B). However, similar results were not detected in HEK293 cells (p>0.05, Fig. 9B) and the counterparts were 0.86±0.09%, 0.97±0.27%, 0.74±0.20% and 0.93±0.25%, respectively.

Cell invasion assay displayed that the transmembrane Hep2 cells in MYCT1-TV-GFP or MYCT1-GFP group were significantly fewer than those in blank and GFP groups (p<0.01, Fig. 9C). The numbers of invasive Hep2 cells in blank, GFP, MYCT1-TV-GFP and MYCT1-GFP groups were 31.33±1.67, 28.33±1.28, 11.50±0.76 and 13.83±1.45, respectively. Moreover, there was no obvious difference between MYCT1-TV-GFP and MYCT1-GFP groups (p>0.05, Fig. 9C). Meanwhile similar results were not observed in HEK293 cells (p>0.05, Fig. 9C) and the counterparts were 12.33±1.45, 10.00±0.58, 8.67±1.76 and 9.33±1.20, respectively.

The results above imply that *MYCT1-TV* and *MYCT1* have the abilities in depressing proliferation and invasiveness, and promoting apoptosis in Hep2 cells, but not in HEK293 cells.

## Discussion

*c-Myc*, together with *L-My*c, and *N-Myc* in the family of *c-Myc* genes, was discovered as the cellular homolog of the retroviral v-myc oncogene 30 years ago [15]. It is also known as a transcription factor through forming heterodimers with its binding partner Max either binding to the canonical E-box sequence CACGTG or to several other noncanonical E-box motifs, such as CATGTG, CATGCG, CACGCG, CACGAG, CGCGAG and CAACGTG [11]–[14]. Dysregulated expression or function of c-Myc is one of the most common abnormalities in human malignancy [16], such as breast cancer [17], colorectal cancer [18], [19], prostate cancer [20], [21], and hepatocellular carcinoma [22], [23]. Overexpression of the c-Myc oncogene has also been observed in LSCC [5], [24]. In mammalian cells, c-Myc expression is highly regulated and closely tied to cell proliferation, growth, apoptosis and differentiation [16], [22].

Given the functions of c-Myc in various biological processes, identification of c-Myc molecular targets has become a hotspot in molecular genetics and oncology research. To date, a large number of c-Myc target genes which could recapitulate c-Myc functions have been confirmed, such as *Cdk4, HMG-I/Y, CAD, Cdc25A, ODC, LDH-A, rcl, Gadd45, P53*, *Tmp* and *mt-mc1* [12], [25]–[28]. For example, *mt-mc1*, *rcl* and *LDH-A* can promote cell transformation and tumorigenesis [28], [29], *mt-mc1*, *Cdc25A, P53* and *Gadd45* can induce apoptosis [28], [30]–[32], *P53*, *Gadd45* and *Cdk*4 can arrest cell cycle [31]–[33]. These genes have one common feature, in which they all have E-box elements in their promoter regions for c-Myc binding.

Our previously study characterized that there are two putative E-box elements in the 5′ flanking sequence of *MYCT1* [9], but it has not been proved by experiment. To address this question, we cloned a 864 bp promoter ranges from position −852/+12 relative to the identified TSS. This only TSS is located at 140 bp upstream of the ATG start codon of *MYCT1-TV*. Luciferase reporter assays showed that this promoter (−852/+12) which efficiently drove luciferase expression in transiently transfected Hep2 and HEK293 cells was functional and exhibited greater transcriptional activity in the presence of two E-box elements. The consistent transcriptional activities of this 864 bp promoter in tumor and normal cells we tested indicated that ubiquitous E-box elements are involved in regulation of *MYCT1-TV* gene transcription. EMSA and ChIP assays showed that c-Myc protein extracted from Hep2 or HEK293 cells can specifically bind to either E-box element between −852 bp and −667 bp region. Furthermore, transcriptional activity of *MYCT1-TV* promoter can be reduced either by site-directed mutagenesis or by c-Myc siRNA transfection, which means c-Myc can specifically enhance *MYCT1-TV* promoter activity. These results provide the support that c-Myc is the important transcription factor for *MYCT1-TV* promoter.

By bioinformatics analysis we found that MYCT1-TV encodes a 187 amino acid protein which is part of 235 amino acid protein encoded by MYCT1 and the shorter 48 amino acid protein contains no obviously structural or functional motif. Since *MYCT1-TV* and *MYCT1* have no structural difference, we are interested in whether they have functional differences. We then detected the expression and biological variation of *MYCT1-TV* and *MYCT1* by RT-PCR, cell proliferation, apoptosis and invasion assays.

Invasion and metastasis are the characteristics of malignant tumours, which are responsible for the primary cause of death in cancer patients. The migration and invasion mechanisms of cancer cells begin with the growth of cancer cells at the primary site of development, followed by decreased local invasion through the surrounding tissue. The larger the primary tumour grows, the more likely that the tumour will be able to acquire traits that lead to a metastatic phenotype. Therefore, inhibition of the invasion and metastasis of tumour cells is a crucial step which would be a useful pathway in the treatment of cancer patients [34]–[39]. Our present study showed that overexpression of *MYCT1-TV/MYCT1* in Hep2 cells could promote cells apoptosis and inhibit cells proliferation and invasion. We speculated this *MYCT1-TV/MYCT1* properties in inhibiting the invasiveness of Hep2 cells probably result from their function on promotion of apoptosis and inhibition of proliferation. In other words, the increased proliferation in Hep2 cells by suppressing cells apoptosis may promote the migration and invasion abilities of Hep2 cells. However, both *MYCT1-TV* and *MYCT1* displayed lower expression in cancer tissues than that in adjacent normal tissues. We also found that *MYCT1-TV* and *MYCT1* do not affect the proliferation, apoptosis and invasion of HEK293 cells. These findings imply that *MYCT1-TV* and *MYCT1* display TSGs-like properties and the down-regulation of them in LSCC perhaps bring about increased proliferation, decreased apoptosis and stepped up invasiveness of tumour cells which may facilitate laryngeal carcinogensis.

The oncogene c-Myc which is overexpressed in LSCC [5], [24] can specifically activate *MYCT1-TV* and *MYCT1* promoter activities, but *MYCT1-TV* and *MYCT1* which appear TSGs-like properties are down-regulated in LSCC. Thus we inferred that *MYCT1-TV/MYCT1* may silence through *MYCT1-TV/MYCT1* promoter methylation in the absence of c-Myc overexpression, which is associated with cancer progression. Alternatively, other transcription factors could be involved in *MYCT1-TV/MYCT1* regulation. This may be the reason for the lower expression and TSGs-like properties of *MYCT1-TV/MYCT1* in LSCC tissues or cells. The specific mechanism need to be further verified.

Results from cells viability and apoptosis assays demonstrated that the overexpression of *MYCT1-TV/MYCT1* could decrease proliferation of Hep2 cells, but increase apoptosis of Hep2 cells. Given these findings, we suggested that as candidate c-Myc target genes, *MYCT1-TV* and *MYCT1* really have some of the known phenotypic features associated with *c-Myc*, but whether they have the other properties of c-Myc and the precise mechanism between c-Myc and *MYCT1-TV/MYCT1V* need to be further studied.

As newly discovered genes, *MYCT1-TV* and *MYCT1* showed no significant differences in the expression or biological function in LSCC or cells. Maybe both of them should be the main forms for *MYCT1* to achieve its function efficiently. At present, the role of *MYCT1-TV* and *MYCT1* is unceasingly studied. Our present study provides a fundamental clue for the further exploration on understanding the function and the molecular mechanism of *MYCT1-TV/MYCT1* in LSCC.

## Materials and Methods

### Cell culture

The human laryngeal cancer cells Hep2, the human hepatoma cells Bel7402, the human epithelial carcinoma cells HeLa, the human gastric carcinoma cells BGC823, SGC7901 and MKN1, and the human gastric epithelial cells GES1 were grown in complete RPMI 1640 medium containing 10% fetal bovine serum. The human embryonic kidney cells HEK293 were maintained in Dulbecco' s high glucose modified Eagle' s medium (DMEM) containing 10% fetal bovine serum. All the cells above were purchased from Cell Biology Institute of Shanghai, Chinese Academy of Science.

### Samples

Forty cases of LSCC tissues were obtained from patients treated at the Ear, Nose and Throat (E.N.T) department of the 463 Hospital of PLA of China after receiving their written informed consent and the approval of the hospital authorities. No patients had previously received any neoadjuvant treatment, e.g. chemotherapy, before the surgery. The specimens, including cancerous tissues and paired adjacent normal laryngeal tissues typically 4–15 mm in diameter were obtained during diagnostic biopsy or operation, and confirmed by pathologists. The clinical pathological characteristics of the patients were evaluated according to the International Union Against Cancer guidelines. All specimens were frozen immediately after surgery and stored at −80°C. Approval for the study was received from the Ethics Committee of China Medical University. Patient information is shown in Table 2.

**Table 2:** The characteristics of the patients (n = 40).

Characteristic	Response	Cases (%)
Age	<62 y	19 (47.5)
	≥62 y	21 (52.5)
Sex	Male	28 (70.0)
	Female	12 (30.0)
Cancer stage	I	7 (17.5)
	II	8 (20.0)
	III	10 (25.0)
	IV	15 (37.5)

### Rapid Amplification of cDNA Ends (RACE)

The 5′ and 3′ end of the gene was elucidated by RACE-PCR using the SMART™ RACE cDNA amplification kit (Clontech, Heidelberg, Germany), according to the manufacturer' s protocol. Total RNA was extracted from normal human blood by an RNA extraction kit (Galen Biopharm, Beijing). The first round PCR of 5′ RACE was performed with the Universal Primer A Mix UPM obtained from the kit and the gene-specific primer GSP1 (5′-CAT AGG CAG GTG GGG GCG GTG TT-3′). PCR product was used in a secondary nested PCR with the Nested Universal Primer A NUP obtained from the kit and the nested gene-specific primer NGSP1 (5′-TGG ACC AGT AGT CAG GAC GGC TCA GA-3′). The 3′-cDNA ends were amplified using the first primer pair GSP2 (5′-TAT CCA TGG CAA TCG GGC TGG TAC T-3′) and UPM followed by a nested PCR with the second primer pair NGSP2 (5′-AGT GGC TGT GAA CGT CGA AGC AAC C-3′) and NUP. The obtained new *MYCT1* transcript variant was deposited in GenBank (*MYCT1-TV*, GenBank accession No. GU997693).

### Sequence prediction and analysis of *MYCT1*

A series of bioinformatics softwares were used as follows: BDGP (www.fruitfly.org/seq_tools/promoter.html); Promoter 2.0 (www.cbs.dtu.dk/services/Promoter/); Promoter Scan (www-bimas.cit.nih.gov/molbio/proscan/); ExPASy proteomics server (http://www.expasy.org/tools/dna.html); NCBI blast (http://blast.ncbi.nlm.nih.gov/Blast.cgi).

### Plasmid construction

For cloning of the *MYCT1* promoter-driven reporter plasmid, genomic DNA was prepared from the human blood using the TIANamp Genomic DNA Kit (Tiangen, China). Plasmids used for functional analysis of the *MYCT1* promoter activity were generated using the promoterless luciferase reporter plasmid pGL3-Basic (Promega, USA). Three 5′ deletion constructs of P852 (−852/+12), P799 (−799/+12), and P667 (−667/+12) were generated by PCR amplification method. Three different forward primers contained an internal site for SacI restriction enzyme each and their sequences are: primer 852: 5′-TTT GAG CTC GTG GCC GAG CGC AGT-3′; primer 799: 5′-TTT GAG CTC GCA GGT GGA TCA CTT GAG G-3′; and primer 667: 5′-TTT GAG CTC GCT GAG GCA GGA GAA TCG-3′. The sequence of the reverse primer containing site for HindIII enzyme is 5′-TTT AAG CTT GTT ATT AGC CAT AAT ATC CAC AAG A-3′.

Mutated reporter plasmids were generated by using the Gene Tailor site-directed mutagenesis system (Invitrogen, USA), according to the manufacturer' s instructions using primers designed to replace the E-box A core sequence “CGCATG” with “TTCATG” (named P852-mutA), replace the E-box B core sequence “CACGCG” with “CAAGGG” (named P852-mutB) and replace both E-box core sequences above (named P852-mutAB). The mutated sequences were cloned into the SacI/HindIII site of pGL3-Basic vectors.

### Luciferase and RNAi assays

Cells in 24-well plates were transfected in triplicate using Lipofectamine 2000 (Invitrogen, USA) with 0.8 µg, respectively, of specific plasmids. After 48 h of transfection, cells were harvested in 100 µl of Passive Lysis Buffer (Promega) and luciferase assay was performed using the Dual Luciferase Assay System (Promega, USA) according to the manufacturer' s instructions. Relative luciferase activity was calculated as the ratio of sample luciferase activity to renilla luciferase activity. Values are expressed as fold changes versus pGL3-Basic.

The c-Myc translation was silenced in Hep2 and HEK293 cells using the siRNA duplexes c-Myc siRNA (5′-GUG CAG CCG UAU UUC UAC UTT-3′) synthesized by Gene Pharma. A nonspecific siRNA (5′-UUC UCC GAA CGU GUC ACG UTT-3′, Gene Pharma) was used as negative control (NC). Cells were transfected with 20 pmol of siRNA, 0.2 µg of P852, P799 or empty construct of the luciferase reporter gene, and 16 ng pRL-TK were cotransfected into cells using Lipofectamine 2000 (Invitrogen, USA) following the manufacturer's instructions. The results of gene knockdown were determined by real time quantitative reverse transcription PCR (qRT-PCR) using VeriQuest™ SYBR Green (USB) and luciferase reporter activity was determined at 48 h after transfection.

### Electrophoretic mobility shift assay (EMSA)

Nuclear extracts were prepared from Hep2 and HEK293 cells using a nuclear extract kit (Active Motif, USA) following the manufacturer's instructions. The following double-stranded oligonucleotides were used (wild type and mutant binding sites are underlined): E-box A, 5′-CGA GCG CAG TGG CGC ATG GCT GTA ATC CCA-3′; E-box B, 5′-CGG GTG TGG TGG CAC GCG CCT GTA ATC CCA-3′; E-box A mutant, 5′-CGA GCG CAG TGG CAT GGG GCT GTA ATC CCA-3′; E-box B mutant, 5′-CGG GTG TGG TGG CGT TAG CCT GTA ATC CCA-3′. Oligonucleotide labeling was performed using the Biotin 3′ End Labeling Kit (Pierce, USA). EMSA used the Lightshift Chemiluminescent EMSA kit (Pierce, USA) according to the protocols provided. For competition assays, samples were preincubated with a 100-fold excess of the unlabeled wild type or mutated oligonucleotide duplex competitors. For the supershift reaction, 1 µg of each anti-c-Myc antibody (N-262X, Santa Cruz) was preincubated with the nuclear extracts in the absence of poly (dI·dC) for 1 hour at 4°C. Subsequently, poly (dI·dC) was added and incubated for 5 min, followed by the addition of a probe for an additional 20 min. Protein DNA complexes were separated by electrophoresis on a 6% non-denaturing acrylamide gel in 0.5×TBE, transferred to positively charged nylon membranes, and visualized by streptavidin-horseradish peroxidase followed by chemiluminescent detection (Pierce, USA).

### Chromatin immunoprecipitation assay (ChIP)

ChIP assay were performed as previously described [40], [41]. Cells were fixed with 1% formaldehyde in growth medium at 37°C for 10 min. After washed with ice-cold PBS, cells were lysed with lysis buffer containing protease inhibitors and sonicated to obtain DNA fragments. Small amount of DNA fragments was saved as input DNA. The rest was diluted, treated with anti-c-Myc antibody (N-262X, Santa Cruz) or rabbit IgG, and immunoprecipitated by protein A/G plus-agarose (Santa Cruz, USA). Each precipitated DNA was eluted and extracted with phenol-chloroform and subjected for PCR. PCR was performed with *MYCT1* promoter-specific primers amplifying the c-Myc binding regions (c-Myc A, forward, 5′-GTT TTC CCT CCT TGA TTT-3′, and reverse, 5′-GTG ATC CAC CTG CTT TG-3′; c-Myc B, forward, 5′-GAG GTC AGG CCT AGT TCA TG-3′, and reverse, 5′-CTT AGT CTC GCT CTG TCG C-3′).

### Construction of MYCT1-TV-GFP and MYCT1-GFP expression vectors

The entire open reading frames of *MYCT1-TV* and *MYCT1* complementary DNA (cDNA) were obtained by RT-PCR from normal human blood. The associated primer sequences are as follows: MYCT1-TV-GFP, forward, 5′-TTT GTC GAC ATG GCT AAT AAC ACA ACA AGT TTA G-3′, and reverse, 5′-TTT GGA TCC TCA GGA ATC TGG GAA TGC C-3′; MYCT1-GFP, forward, 5′-TTT GTC GAC ATG CGA ACA CAA GTA TAT GAG GGG T-3′, and reverse, 5′-TTT GGA TCC TCA GGA ATC TGG GAA TGC C-3′. Two different forward primers contains a SalI site and two different reverse primers contains a BamHI site. The PCR fragments were cloned into the SalI/BamHI site of pEGFP-C1 plasmids (BD, USA). The fidelity of the constructs was then confirmed by sequencing, and plasmids were prepared for transfection using TIANpure Mini Plasmid Kit (Tiangen, China). MYCT1-TV-GFP, MYCT1-GFP and pEGFP-C1 vectors (as a negative control) were then transfected into Hep2 cells and HEK293 cells using Lipofectamine 2000 (Invitrogen, USA) following the manufacturer' s instructions.

### Semi-quantitative reverse transcription-polymerase chain reaction (RT-PCR)

Total RNA was isolated from Bel7402 cells, Hep2 cells, HeLa cells, BGC823 cells, SGC7901 cells, MKN1 cells, GES1 cells, HEK293 cells, human blood, LSCC tissues and paired adjacent normal laryngeal tissues using Trizol reagent (Invitrogen, USA) following the protocol of the manufacturer. cDNA was synthesized by reverse transcription using an AMV RNA PCR kit (TaKaRa, China) in keeping with the standard operating procedure. The *MYCT1* primers were the same as those for construction of MYCT1-GFP, which were expected to produce a 726 bp DNA fragment. The primers for *MYCT1-TV* were 5′- TAA TAA CAC AAC AAG TTT AGG GAG T and 5′-AGT CAC ATT GGA AGT AAA TGA GGA TTG-3′, which were expected to produce a 929 bp DNA fragment. β-actin served as an internal control in each sample. The primer sequences for β-actin were 5′-CTC TTC CAG CCT TCC TTC CT-3′ and 5′-CAC CTT CAC CGT TCC AGT TT-3′, which were expected to produce a 511 bp DNA fragment. The electrophoresis images were scanned by Fluor-S MultiImager (Bio-Rad, USA) and the original intensity of each specific band was quantified with the software Multi-Analyst (Bio-Rad, USA). Data were analyzed after being normalized by the intensity of β-actin.

### Cell viability assay

Cell viability was assessed by the MTT colorimetric assay. 50 mg of MTT (Sigma, USA) was dissolved in 10 mL of PBS. After being seeded for 24 h in a 96-well plate, Hep2 and HEK293 cells (1×10^4^ cells/well) were transfected with GFP only, MYCT1-TV-GFP and MYCT1-GFP for 48 h, with untransfected cells serving as a control. At 48 h after transfection, 10 µl of MTT solution (5 mg/mL) was added to each well. After incubation for 4 h at 37°C, the medium was replaced with 200 µl of DMSO and the plate was allowed to shake on a plate shaker for 10 min. Absorbance at 490 nm was measured using a microplate reader, and the results were expressed as a percentage (%) of the control. All experiments were done with three parallel wells each and repeated three times [42].

### Apoptosis assay

Apoptotic cells were measured by using an annexin V-PE/7-AAD Kit (KeyGEN, China) according to the manufacturer's protocol. The apoptotic rates were analysed by flow cytometry on a FACS Calibur (Becton Dickinson, USA). Cells that were annexin V-PE positive and 7-AAD negative indicated early apoptotic cells [42].

### Transwell chamber invasion assay

Twenty-four-well invasion chambers were obtained from Costar (USA). After being transfected with negative control GFP, MYCT1-TV-GFP, and MYCT1-GFP, Hep2 (2×10^5^ cells) and HEK293 (4×10^5^ cells) cells were detached and resuspended in serum-free medium, and seeded to the upper chambers. 500 µl of medium containing 10% FBS was added to the lower chambers. After incubation at 37°C for 24 h, cells remaining attached to the upper surface of the filters were carefully removed with cotton swabs. The filters were fixed with methanol and stained with haematoxylin and eosin. The cells that invaded to the underside of the filter were counted [43].

### Statistics

Data were subjected to statistical analysis and shown as mean ± standard error of the mean (SEM). Differences in mean values were analyzed using one-way analysis of variance (ANOVA). Comparisons related to age or sex in clinical characteristics were made by the Mann-Whiney U test. The comparisons of mRNA levels between *MYCT1-TV* and *MYCT1* in LSCC and paired adjacent normal laryngeal tissues were made by Levene's test. In the figures, the statistically significant values were marked with asterisks (*, p<0.05; **, p<0.01).
